# Rotavirus group A genotype circulation patterns across Kenya before and after nationwide vaccine introduction, 2010–2018

**DOI:** 10.1186/s12879-020-05230-0

**Published:** 2020-07-13

**Authors:** Mike J. Mwanga, Betty E. Owor, John B. Ochieng, Mwanajuma H. Ngama, Billy Ogwel, Clayton Onyango, Jane Juma, Regina Njeru, Elijah Gicheru, Grieven P. Otieno, Sammy Khagayi, Charles N. Agoti, Godfrey M. Bigogo, Richard Omore, O. Yaw Addo, Seheri Mapaseka, Jacqueline E. Tate, Umesh D. Parashar, Elizabeth Hunsperger, Jennifer R. Verani, Robert F. Breiman, D. James Nokes

**Affiliations:** 1 Wellcome Trust Research Programme, Kenya Medical Research Institute, Kilifi, Kenya; 2 Kenya Medical Research Institute, Center for Global Health Research (KEMRI-CGHR), Kisumu, Kenya; 3 Division of Global Health Protection, US Centers for Disease Control and Prevention, Nairobi, Kenya; 4 Global Health Institute, Emory University, Atlanta, GA USA; 5 Department of Virology, South African Medical Research Council/Diarrheal Pathogens Research Unit, Sefako Makgatho Health Sciences University, Pretoria, South Africa; 6 Division of Viral Diseases, US Centers for Disease Control and Prevention, Atlanta, GA USA; 7 School of Life Science, and Zeeman Institute for Systems Biology and Infectious Disease Epidemiology Research (SBIDER), University of Warwick, Coventry, CV47AL UK

**Keywords:** Rotavirus, Genotype, Pre-vaccine, Post-vaccine, Kenya

## Abstract

**Background:**

Kenya introduced the monovalent G1P [8] Rotarix® vaccine into the infant immunization schedule in July 2014. We examined trends in rotavirus group A (RVA) genotype distribution pre- (January 2010–June 2014) and post- (July 2014–December 2018) RVA vaccine introduction.

**Methods:**

Stool samples were collected from children aged < 13 years from four surveillance sites across Kenya: Kilifi County Hospital, Tabitha Clinic Nairobi, Lwak Mission Hospital, and Siaya County Referral Hospital (children aged < 5 years only). Samples were screened for RVA using enzyme linked immunosorbent assay (ELISA) and VP7 and VP4 genes sequenced to infer genotypes.

**Results:**

We genotyped 614 samples in pre-vaccine and 261 in post-vaccine introduction periods. During the pre-vaccine introduction period, the most frequent RVA genotypes were G1P [8] (45.8%), G8P [4] (15.8%), G9P [8] (13.2%), G2P [4] (7.0%) and G3P [6] (3.1%). In the post-vaccine introduction period, the most frequent genotypes were G1P [8] (52.1%), G2P [4] (20.7%) and G3P [8] (16.1%). Predominant genotypes varied by year and site in both pre and post-vaccine periods. Temporal genotype patterns showed an increase in prevalence of vaccine heterotypic genotypes, such as the commonly DS-1-like G2P [4] (7.0 to 20.7%, *P* < .001) and G3P [8] (1.3 to 16.1%, *P* < .001) genotypes in the post-vaccine introduction period. Additionally, we observed a decline in prevalence of genotypes G8P [4] (15.8 to 0.4%, *P* < .001) and G9P [8] (13.2 to 5.4%, *P* < .001) in the post-vaccine introduction period. Phylogenetic analysis of genotype G1P [8], revealed circulation of strains of lineages G1-I, G1-II and P [8]-1, P [8]-III and P [8]-IV. Considerable genetic diversity was observed between the pre and post-vaccine strains, evidenced by distinct clusters.

**Conclusion:**

Genotype prevalence varied from before to after vaccine introduction. Such observations emphasize the need for long-term surveillance to monitor vaccine impact. These changes may represent natural secular variation or possible immuno-epidemiological changes arising from the introduction of the vaccine. Full genome sequencing could provide insights into post-vaccine evolutionary pressures and antigenic diversity.

## Background

Childhood diarrhea caused by rotavirus group A (RVA) infection remains a leading cause of morbidity and mortality in young children globally [1]. In 2016, RVA infections were estimated to be responsible for 1,537,000 hospitalization cases, 128,500 deaths globally and over 80% of these deaths occurred in developing countries [2]. In Kenya alone, it is estimated that rotavirus infection accounts for over 3000 deaths annually in children under 5 years of age [3].

Upon infection by the virus, immune response to RVA by the host is directed to the highly variable VP7 and VP4 genes found on two separate segments of the double-stranded RNA genome [4]. RVA G and P genotypes exist as multiple variants in nature, few of which have been found to infect humans [4]. Up to 36 G and 51 P genotypes have been detected globally in both humans and animals, with multiple G-P combinations [5]. Molecular studies have characterized circulating genotypes worldwide with predominance of genotypes G1P [8], G2P [4], G3P [8], G4P [8], G9P [8] and G12P [8] (in decreasing order of prevalence) [6, 7]. Although the distribution of these genotypes varies from region to region and from one season to another, genotype G1P [8] has remained the most dominant genotype globally [6]. In Africa, there is a high diversity of genotypes, most commonly G1P [8], G2P [4], G9P [8], G2P [6], G12P [8] and G3P [6], with G1P [8] and G2P [4] predominant [7, 8]. Significant RVA infections are also caused by strains of uncommon genotypes including G1P [4], G2P [8], G9P [4], G12P [4], G8P [6], G8P [8] and G12P [6] [7, 8]. Such uncommon strains also show a wide variation from one region to the other, and can spread globally to become common strains. For instance, genotypes G9P [8] and G12P [8] emerged and contributed to a larger proportion of global RVA infections [9]. An understanding of these genetic diversity after vaccine introduction is necessary for design and implementation of effective control programs.

In 2009, the World Health Organization (WHO) recommended the inclusion of RotaTeq® (Merck Vaccines, Whitehouse Station, New Jersey) or Rotarix® (GlaxoSmithKline Biologicals, Rixensart, Belgium) vaccines in the national immunization programs of countries that experience high diarrhea morbidity and mortality burden due to RVA disease [10]. The introduction and increased use of these vaccines have reduced up to 76% of rotavirus hospitalizations in children < 5 years [11] and averted up to 28,800 deaths globally, including 84% of the deaths in sub-Saharan Africa [2]. Furthermore, these vaccines showed overall good clinical protection against multiple homotypic and heterotypic RVA strains in humans [12]. Kenya incorporated the monovalent G1P [8] Rotarix® vaccine into the national immunization program in July 2014, administered in two oral doses offered at weeks 6 and 10 of age. The Rotavirus Immunization Programme Evaluation in Kenya (RIPEK) was established as a collaboration among existing rotavirus surveillance platforms across Kenya to monitor the impact of Rotarix vaccine introduction against rotavirus disease and circulating RVA genotypes. Substantial effectiveness of the vaccine in Kenya (vaccine coverage of 72% [13]) and the entire sub-Saharan region (where disease burden is high) has been recorded, and the decline in incidence of all-cause and rotavirus associated diarrhea admissions has been attributed to vaccine implementation [14–18]. However, there are limited data on RVA diversity in post-vaccine introduction periods in this region. The current report describes the distribution and temporal patterns of RVA genotypes observed before and after Rotarix vaccine introduction in Kenya.

## Methods

### Rotavirus surveillance

RVA surveillance was carried out in four health facilities in coastal, western and central regions of Kenya. These surveillance sites were: Kilifi County Hospital (KCH) in Kilifi County, Tabitha Clinic (TC), in Kibera, Nairobi County, Saint Elizabeth Lwak Mission Hospital (LMH) and Siaya County Referral Hospital (SCRH) in Siaya County (Supplementary Figure 1). KCH is a government hospital located on the Kenyan coast serving a rural and semi-rural population. Rotavirus surveillance at KCH started in September 2009, and is implemented by KEMRI Wellcome Trust Research Program (KWTRP) a partnership between Kenya Medical Research Institute and Wellcome Trust, UK [19, 20]. SCRH is a government hospital which also serves a rural and semi-rural population in Siaya County, Western Kenya. SCRH has had an active rotavirus surveillance since 2010 and is implemented by KEMRI Center for Global Health Research, KEMRI-CGHR in collaboration with US Centers for Disease Control and Prevention (CDC) [21]. LMH is a private health facility serving a rural population in Asembo, Siaya County where rotavirus surveillance is carried out as part of the Population Based Infectious Disease Surveillance (PBIDS) platform under KEMRI-CGHR and CDC [22]. PBIDS also supports rotavirus surveillance at Tabitha Clinic, a private health facility in Kibera, Nairobi County.

Surveillance was conducted during January 2010 – December 2018 for all the sites except for SCRH where surveillance ended in December 2016. Stool samples were collected from children aged < 13 years (in KCH, TC, LMH) and < 5 years (in SCRH) of age presenting with acute gastroenteritis (AGE). AGE was defined as ≥3 watery stools passed within a 24-h period during the illness for KCH, TC and LMH, while for SCRH, AGE was defined as ≥3 loose stools and/or ≥ 1 episode of unexplained vomiting followed by loose stool within a 24-h period beginning no more than 7 days before the visit to SCRH.

### Laboratory processing

RVA was tested by use of commercially available enzyme immunoassays. The ProSpecT™ Rotavirus Kit (Oxoid, Basingstoke UK) was used to test samples collected from KCH while the Rotaclone® kit (Premier™ Meridian Bioscience, Cincinnati, Ohio, USA) was used to test samples collected from LMH, SCRH, and TC. For samples collected from LMH, KCH and TC, partial fragments of the segments encoding the outer capsid proteins, VP4 (660 bp) and VP7 (881 bp), were amplified in a One-step Reverse Transcriptase Polymerase Chain Reaction (RT-PCR) using previously described primer pairs [23, 24]. Successful amplification was visualized by electrophoresis of the PCR product in a 2% agarose gel. PCR products of confirmed positives were purified using GFX DNA purification kit (GFX-Amersham, Amersham, UK), according to the manufacturer’s instructions. Confirmed positives were then sequenced using Big Dye Terminator 3.1 (Applied Biosystems, Foster City, California, USA) with the same primers as in PCR amplification on an ABI Prism 3130xl Genetic Analyzer (Applied Biosystems, Foster City, California, USA).

### RVA genotyping

Reads from the sequencer were trimmed (removing regions including primer sequences) and assembled into contigs (consensus sequence formed from aligning the forward and reverse reads) using Sequencher version 5.4.6 (Gene Codes Corp Inc., Ann Arbor, MI, USA). The cleaned sequences for the VP7 and VP4 genes have been deposited to GenBank database: https://www.ncbi.nlm.nih.gov/genbank/. Sequence accession numbers for these genes are provided in supplementary Table 2 and supplementary Table 3, respectively. G and P genotypes were determined by submitting cleaned sequences to the online automated Virus Pathogen Resource genotyping tool [25]. Using this method only a single genotype per specimen was identified. However, 8.6% (*n* = 113) of the samples were typed for only one of the genes due to failure in sequencing and/or contig assembly, supplementary Table 1, and were excluded from the main analysis. For positive samples collected from SCRH, sample processing and genotyping was performed as previously described [26]. Briefly, full length VP7 gene (1062 bp) was amplified using sBeg9 and End9 primers. The resulting cDNA was used for G typing using primers End9, aAT8v, aBT1, aCT2, aDT4, mG3, mG9, mG10 and G12 for G1, G2, G3, G4, G8, G9, G10 and G12 genotypes, respectively [27, 28]. Alternatively, partial-length amplification of the VP4 gene (876 bp) was achieved by use of Con2 and Con3 primers. P typing was performed using a mixture of primers consisting of Con3 and primers 1 T-1, 2 T-1, 3 T-1, 4 T-1, 5 T-1, mP [11] and P [14] for genotypes P [8], P [4], P [6], P [9], P [10], P [11] and P [14], respectively [23, 29]. In this instance, untypable G (8.9%, *n* = 15/168) and P (22.6%, *n* = 38/168) genotypes and mixed infections (18.5%, *n* = 31/168) were observed and subsequently excluded from analysis.

### Phylogenetic analysis of the Kenyan G1 and P [8] vaccine strains

Maximum likelihood (ML) method was used to determine the phylogenetic relationship of the partial sequences of genotypes G1 and P [8] observed during the pre- and post-vaccine periods in Kenya, and further compared to RVA strains circulating globally. Global contemporaneous sequences were retrieved from GenBank (as of May 2020) and compared to local strains. Sequences were aligned using MAFFT v7.2 [30] and visualized in AliView v1.8 [31]. Best-fit volutionary models were tested and selected in IQTREE v1.6 [32] using the Bayesian Information Criteria [33]. ML trees were inferred using IQTREE with 1000 bootstrap replicates. The resulting trees were visualized and edited in FigTree v1.4.3 (http://tree.bio.ed.ac.uk/software/figtree/). Nucleotide distances matrixes were prepared using the p-distance algorithm inferred in MEGA v10 [34]. The trees were drawn to scale indicating nucleotide substitutions rates per site.

### Data analysis

Data collating and analyses were performed in Microsoft Excel and R version 3.5. Genotype data collected from LMH and SCRH were merged to constitute data from Siaya County. The summary of proportions was conducted for each genotype by site and period of detection. Frequency distribution and temporal pattern graphs were generated. Two-sided proportional tests of the most common genotypes during the pre-vaccine (January 2010 thru June 2014) and post-vaccine (July 2014 thru December 2018) eras were performed in R, and *P*-values < 0.05 were considered statistically significant.

## Results

A total of 6562 stool samples were collected from health facilities in LMH, SCRH, TC and KCH. Of the 1312 (20.0%) RVA positive samples, individual G and P genotypes were identified in 928 (70.7%) and 904 (68.9%) samples, respectively, while 875 (66.7%) samples were successfully genotyped for both G and P genes [LMH (*n* = 38), SCRH (*n* = 161), TC (*n* = 84), and KCH (*n* = 592)]. Of these, 614 (70.2%) were observed in the pre-vaccine (January 2010 – June 2014) and 261 (29.8%) in the post-vaccine (July-2014 – December 2018) introduction periods, respectively.

### G and P genotypes circulating in Kenya

Overall, nine different G types (G1, G2, G3,G4, G8, G9, G10, G12, G29) and four P types (P [4], P [6], P [8] and P [14]) were observed in Kenya. The most common G type was G1 (49.5%) followed by G9 (12.7%), G8 (12.0%), G2 (11.1%) and G3 (10.1%), Table 1. Genotypes G4, G10, G12 and G29 were detected at low frequencies (< 4%). The most common P types were P [8] (68.4%) and P [4] (23.6%), while P [6] (7.7%) and the less common P [14] (0.3%) were also observed, Table 1.

**Table 1:** Frequency of RVA G and P genotype circulation in Kenya between 2010 and 2018

G Genotypes	n	%	P Genotypes	n	%
G1	459	49.5	P [4]	213	23.6
G2	103	11.1	P [6]	70	7.7
G3	94	10.1	P [8]	618	68.4
G4	1	0.1	P [14]	3	0.3
G8	111	12.0			
G9	118	12.7			
G10	5	0.5			
G12	36	3.9			
G29	1	0.1			
Total	928		Total	904	

The table represents number of samples where only one genotype was observed. Proportion of specimen observed as mixed infections is indicated under section 2.3

### Rotavirus genotype distribution in pre- and post- vaccination eras

In the entire period, 22 different G/P combinations were identified, Table 2. G1P [8] (47.7%) was the most common genotype followed by G8P [4] (11.2%), G2P [4] (11.1%), G9P [8] (10.9%), G3P [8] (5.7%) and G3P [6] (2.9%). In addition to these common genotypes, multiple other rare genotypes; G1P [4], G1P [6], G1P [14], G2P [8], G3P [4], G3P [6], G4P [4], G8P [6], G8P [14], G9P [6], G9P [14], G10P [8], G12P [4], G12P [6], G12P [8] were observed in low frequency (< 2%), Table 2.

**Table 2:** Temporal distribution of RVA GP genotypes observed in Kenya between 2010 and 2018

	2010		2011		2012		2013		2014		2015		2016		2017		2018		Total	
Genotype	n	%	n	%	n	%	n	%	n	%	n	%	n	%	n	%	n	%	n	%
G1P [8]	40	26.1	117	73.1	39	26	60	57.7	38	46.9	91	90.1	8	18.2	7	43.8	17	25.8	417	47.7
G8P [4]	54	35.3	0	0	42	28	1	1	0	0	0	0	0	0	1	6.3	0	0	98	11.2
G2P [4]	11	7.2	1	0.6	19	12.7	5	4.8	14	17.3	3	3	35	79.5	5	31.3	4	6.1	97	11.1
G9P [8]	18	11.8	20	12.5	30	20	8	7.7	17	21	1	1	0	0	0	0	1	1.5	95	10.9
G3P [8]	0	0	0	0	0	0	4	3.8	4	4.9	0	0	0	0	1	6.3	41	62.1	50	5.7
G3P [6]	2	1.3	0	0	2	1.3	13	12.5	2	2.5	4	4	0	0	2	12.5	0	0	25	2.9
G12P [6]	2	1.3	5	3.1	4	2.7	1	1	1	1.2	0	0	0	0	0	0	1	1.5	14	1.6
G1P [6]	1	0.7	1	0.6	2	1.3	9	8.7	0	0	0	0	0	0	0	0	0	0	13	1.5
G12P [8]	2	1.3	6	3.8	1	0.7	0	0	0	0	1	1	0	0	0	0	1	1.5	11	1.3
G9P [6]	8	5.2	1	0.6	1	0.7	0	0	1	1.2	0	0	0	0	0	0	0	0	11	1.3
G1P [4]	5	3.3	1	0.6	2	1.3	0	0	0	0	1	1	0	0	0	0	0	0	9	1
G9P [4]	4	2.6	2	1.3	1	0.7	0	0	0	0	0	0	0	0	0	0	0	0	7	0.8
G10P [8]	0	0	4	2.5	0	0	1	1	1	1.2	0	0	0	0	0	0	0	0	6	0.7
G8P [8]	1	0.7	1	0.6	3	2	0	0	0	0	0	0	0	0	0	0	0	0	5	0.6
G8P [6]	1	0.7	0	0	3	2	0	0	0	0	0	0	0	0	0	0	0	0	4	0.5
G2P [8]	1	0.7	0	0	0	0	1	1	1	1.2	0	0	0	0	0	0	1	1.5	4	0.5
G12P [4]	2	1.3	1	0.6	0	0	0	0	0	0	0	0	0	0	0	0	0	0	3	0.3
G3P [4]	0	0	0	0	0	0	0	0	2	2.5	0	0	0	0	0	0	0	0	2	0.2
G4P [4]	0	0	0	0	0	0	0	0	0	0	0	0	1	2.3	0	0	0	0	1	0.1
G1P [14]	0	0	0	0	0	0	1	1	0	0	0	0	0	0	0	0	0	0	1	0.1
G9P [14]	0	0	0	0	1	0.7	0	0	0	0	0	0	0	0	0	0	0	0	1	0.1
G8P [14]	1	0.7	0	0	0	0	0	0	0	0	0	0	0	0	0	0	0	0	1	0.1
Total	153		160		150		104		81		101		44		16		66		875	

**Fig. 1:**
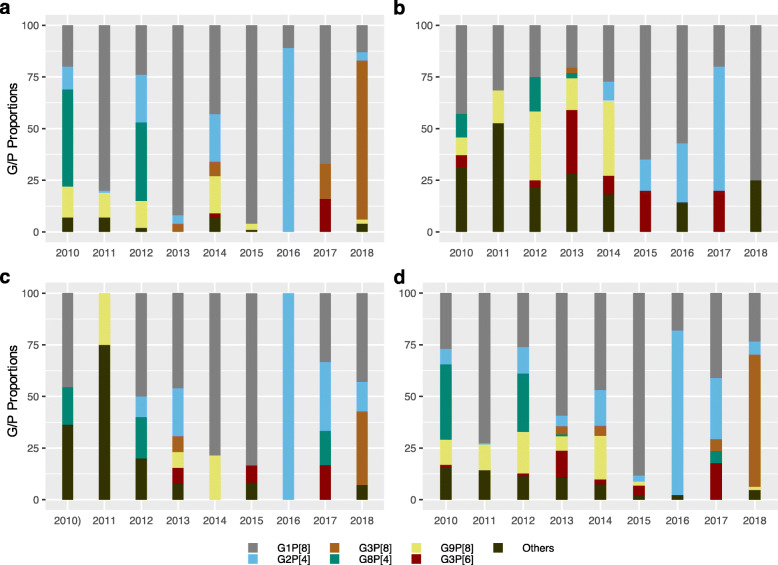
Temporal rotavirus genotype distribution in the three surveillance sites across Kenya; **a** Kilifi County Hospital in Kilifi County, **b** Tabitha Clinic in Kibera, Nairobi County, **c** Siaya County (combined genotype data from Lwak Mission Hospital and Siaya Referral Hospital) and **d** combination of the three Counties in Kenya between 2010 to 2018

Figure 1 a, b, c and d show the temporal distribution of RVA genotypes in Kilifi, Siaya (i.e, pooled SCRH and LMH data) and Nairobi counties, and Kenya (pooled countrywide), respectively. Genotypes G1P [8], G2P [4], G8P [4], G3P [6], G3P [8] and G9P [8] were observed across all three sites. G1P [8] was observed in all periodss, in all sites, except in 2011 and 2016 in Nairobi. In contrast, genotypes G2P [4], G3P [8], G3P [6], G8P [4] and G9P [8] showed a fluctuating pattern in all three sites. Notably, genotype G2P [4] was observed in pre- and post-vaccine periods in Kilifi and Nairobi, and only in the post- vaccine period in Siaya. Furthermore, genotype G3P [8] was observed in Kilifi and Nairobi counties in pre- and post-vaccine periods and only in pre-vaccine period in Siaya, while genotype G3P [6] was detected in all three sites, although in moderate proportions. Additionally, genotype G9P [8] was observed in pre- and post- vaccine periods in Kilifi, unlike in Nairobi and Siaya where it was only observed in the pre-vaccine period. Genotype G8P [4] occurred in pre- and post-vaccine periods in Nairobi county and only in the pre-vaccine period in Kilifi and Siaya counties.

During the period before vaccine introduction in Kenya, G1P [8] (45.8%) was the predominant genotype observed in this population, followed by G8P [4] (15.8%), G9P [8](13.2%), G2P [4] (7.0%) and G3P [6] (3.1%). After vaccine introduction, G1P [8] remained the dominant genotype (52.1%), followed by G2P [4] (20.7%), G3P [8] (16.1%), G9P [8] (5.4%) and G3P [6] (2.7%). Multiple other genotypes were also observed in pre- and post-vaccine periods, although in low proportions (< 2%), Tables 2, 3.

**Table 3:** Frequency of RVA genotypes detected in Kenya in pre-vaccine (2010 - June 2014) and post-vaccine (July 2014–2018)

	Pre-vaccine (2010-June 2014)					Post-vaccine (July 2014–2018)			
	P [4]	P [6]	P [8]	P [14]	Total	P [4]	P [6]	P [8]	Total
G1	8	13	281	1	303	1	0	136	137
G2	43	0	2	0	45	54	0	2	56
G3	2	19	8	0	29	0	7	42	49
G4	0	0	0	0	0	1	0	0	1
G8	97	4	5	1	107	1	0	0	1
G9	7	11	81	1	100	0	0	14	14
G10	0	0	6	0	6	0	0	0	0
G12	3	12	9	0	24	0	2	1	3
Total	160	59	392	3	614	57	9	195	261

Overall, at these surveillance sites, the first four RVA periods, saw an alternating pattern of dominance between G8P [4] and G1P [8] (Fig. 1d). G8P [4] was the predominant genotype in 2010 (54/153; 35.3%) and in 2012 (42/150;28.0%), while G1P [8] predominated in 2011 (117/160;73.1%) and 2013 (60/104;57.7%) through to 2014 (vaccine introduction periods) breaking the cyclic pattern. During this periods, G2P [4] and G9P [8] circulated in moderate proportions (1–20%) and (2–14%), respectively (Table 2). After vaccine introduction, G1P [8] dominated immediately in the first year of vaccine introduction (2015) (91/101, 90.1%). This phenomena was however, short lived and there was a re-emergence of G2P [4] (35/44; 79.5%) in the second year of vaccine period (2016), which was among the common genotypes in 2017 together with genotype G1P [8](7/16;43.6%). G3P [8] (41/66;62.1%) dominated in the fourth year post-vaccine introduction period (2018), whereas G1P [8] (17/66;25.8%) and G2P [4] (4/66;6.1%) continuously circulated, although in reduced proportions. Notably, a separate analysis of the post-vaccine G3P [8] (data not shown), revealed that 4.8% (*n* = 2/41) were of the equine-like genotype while the rest were wild-type genotype.

**Fig. 2:**
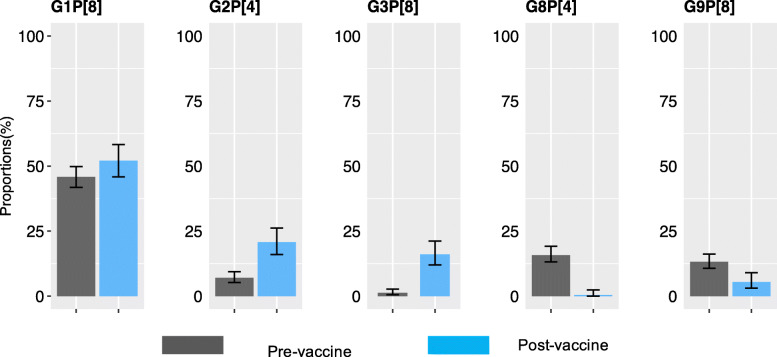
Comparison of prevalence of the dominant genotypes (G1P [8], G2P [4], G3P [8], G8P [4] and G9P [8]) at 95% confidence interval (CI) during the pre- (Jan 2010-Jun 2014) and post-(July 2014 – Dec 2018) vaccine introduction periods in Kenya. The predominant genotypes were selected based on their frequency as indicated in Table 3

In the post-vaccine introduction period, relative to the pre-vaccine period, we observed a significant increase in prevalence of genotypes G2P [4] (7.0% vs. 20.7%, *P* < .001) and G3P [8] (1.3% vs. 16.1%, *P* < .001) and significant decline in prevalence of genotypes G8P [4] (15.8% vs. 0.4%, *P* < .001) and G9P [8] (13.2% vs. 5.4%, *P* < .001), Fig. 2. No significant difference in the prevalence of the vaccine genotype G1P [8] (45.8 vs. 52.1%,*P* = .35) was measured before and after vaccine introduction. A decline in genotype diversity was observed after vaccine introduction (11 genotypes) as compared to pre-vaccine period (21 genotypes).

### Phylogenetic analysis of the Kenya G1 and P [8] vaccine strains

**Fig. 3:**
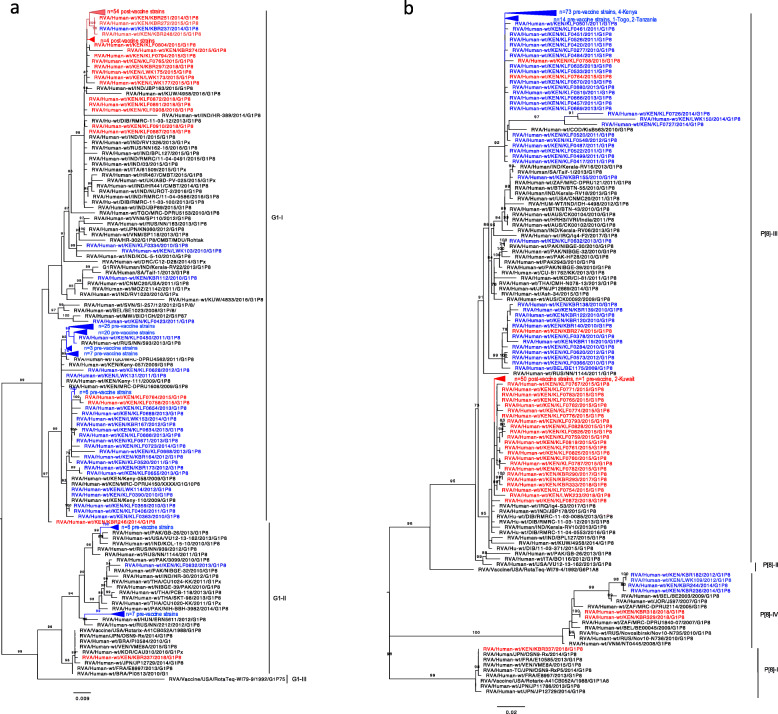
**a** Maximum likelihood phylogenetic tree of representative partial VP7 sequences (*n* = 180) of the G1 genotype that circulated in Kenya between 2010 and 2018. Pre-vaccine Kenyan strains are marked as blue taxa, post-vaccine Kenyan strains are marked as red taxa while global strains retrieved from GenBank are marked as black taxa. Only bootstrap values greater than or equal to 70 are shown. The scale bars indicate nucleotide substitutions per site per year. **b** Maximum likelihood phylogenetic tree of representative partial VP4 sequences (*n* = 209) of the P [8] genotype that circulated in Kenya between 2010 and 2018. Pre-vaccine Kenyan strains are marked as blue taxa, post-vaccine Kenyan strains are marked as red taxa while global strains s retrieved from GenBank are marked as black taxa. Only bootstrap values greater than or equal to 70 are shown. The scale bars indicate nucleotide substitutions per site per year

As shown in Fig. 3a, phylogenetic analysis of Kenyan G1 strains revealed circulation of lineages G1-I and G1-II with representative sequences from all the three sites (Kilifi, Kibera and Lwak). In both pre and post-vaccine periods, detected strains were predominantly found within lineage G1-I with a few within lineage G1-II. Interestingly, within lineage G1-I, pre and post vaccine strains formed separate clusters. However, three post-vaccine strains (2015; *n* = 2, 2014; *n* = 1) clustered within pre-vaccine strains, which showed high nucleotide sequence identities of up to 99% to pre-vaccine strains. Additionally, a single pre-vaccine strain, observed in 2014, grouped within the post-vaccine cluster, which showed high nucleotide identities of up to 100% to other post-vaccine strains. The lineage G1-I strains also clustered with other strains isolated from Kenya (2009), Malawi (2012), Togo (2011), Russia (2013), India (2013), Kuwait (2016), USA (2011) and Italy (2015), with high nucleotide identities ranging between 98 and 99%. Lineage II cluster contained strains observed in pre-vaccine period (*n* = 14) which clustered with other globally isolated strains from Pakistan (2013), India (2012), USA (2013) and Hungary (2012), with nucleotide identities of up to 99%. In contrast, a single post-vaccine strain, isolated in Kibera (KBR337), was observed within lineage G1-II cluster, which showed high nucleotide and amino acid identity of up to 100% to the Rotarix vaccine and other vaccine-derived strains from Japan, Brazil and Korea.

As shown in Fig. 3b, analysis of the Kenyan P [8] genotype revealed groupings into lineage P [8]-III (predominant lineage) with a few others belonging in lineage P [8]-I and P [8]-IV. Within lineage P [8]-III, distinct clusters of pre and post-vaccine strains were evident with clusters containing genotypes from all the three study sites (Kibera, Lwak and Kilifi). However, three post-vaccine strains (observed in 2015) clustered with pre-vaccine strains, while one pre-vaccine strain (observed in 2014) clustered within post-vaccine genotypes. The strains in lineage III clustered with other strains isolated from Kenya (2009), Togo (2011), Congo (2010), Tanzania (2011), India (2011), USA (2011), Pakistan (2010) and Kuwait (2016) showing nucleotide identities ranging from 97 to 98%. The P [8]-I cluster contained a single post-vaccine strain (KBR337), from Kibera, which showed 100% nucleotide and amino acid identity to the Rotarix vaccine and other vaccine-derived strains observed in Vietnam and Japan. Lineage P [8]-IV cluster contained strains observed in pre (2012 and 2014, from Kibera and Lwak) and post (2018, from Kibera) vaccine periods and clustered with isolates from Russia (2010).

## Discussion

Prior to vaccine introduction, G1P [8] was the predominant genotype. However, this genotype’s prevalence varied substantially from year to year (dominant in 2011, 2013, 2014 and 2015; less so in 2010 and 2012), emphasizing the limits of short-term surveillance and the potential for misrepresentation of patterns. During 2010 and 2012, a large proportion of children were infected with genotype G8P [4], which was not detected in any years following vaccination (except during 2017). Genotypes G1P [8], G2P [4] and G9P [8] have also been identified as dominant genotypes in a study involving six countries from Eastern and Southern Africa [7].

Genotype prevalence varied year to year and from before to after vaccine introduction. Interestingly, while genotype G1P [8] showed no overall change in prevalence compared to the pre-vaccine period, it predominated only in the first year after vaccine introduction (2015), and declined in prevalence thereafter, in particular to be replaced by G2P [4] in 2016, and by G3P [8] in 2018. Genotypes G8P [4] and G9P [8], which were the second and third common genotypes in pre-vaccine period, significantly declined in the post-vaccine period. Predominance of G2P [4] and G3P [8] has been reported worldwide following universal Rotarix vaccine introduction. For instance, G2P [4] was observed in Australia [35] (in states using Rotarix) and Belgium [36] after vaccine introduction, while data from Ethiopia [37] and Madagascar [38] depicted an increase in prevalence of G3P [8] in post-vaccine introduction period. Studies on the G3P [8] have revealed the emergence and spread of strains of equine-like G3 genotype co-circulating with wild-type G3 strains [39–41]. In here, 4.8% (2/41) were detected to be of equine-like type (data not shown). Further analysis on this genotype will illuminate of the genetic diversity and prevalence of the emerging equine-like in the post-vaccine introduction period. G2P [4] is one of the genotypes of concern since this fully heterotypic genotype has a different genomic constellation (DS-1-like) to the genotype in the monovalent Rotarix vaccine (Wa-like) [42]. There is no conclusive evidence associating the increase in prevalence of these genotypes to selective vaccine pressure created by implementation of Rotarix monovalent vaccine. Whilst the vaccine offers both homotypic and heterotypic protection [43, 44], the emergence of the fully heterotypic G2P [4] and partially heterotypic G3P [8] genotypes, and persistence in circulation of the homotypic G1P [8] genotype after vaccine introduction emphasizes the need for continued monitoring of impact of vaccine on genotypes.

We observed multiple uncommon G/P combinations including G1P [4], G1P [6], G2P [8],G4P [4], G8P [8],G8P [14], G10P [8], G12P [4] and G12P [6] at frequency of < 2%. Genotype P [14] has been described sporadically in humans and is believed to have originated from animal rotavirus and introduced into humans through interspecies transmission and/or reassortment events [45]. The circulation of these uncommon genotypes demonstrates the high diversity of RVA genotypes in Kenya and concurs with findings from other African countries [7]. However, genotype diversity was seen to decline in post-vaccine period (only 11 GP combinations, compared to 21 in pre-vaccine period), mirroring the experience of other countries, including Brazil and Zimbabwe, which also indicated a decline in genotype variation after vaccine introduction [46, 47]. This is in contrast to other countries such Australia which observed increase in genotypes after vaccine introduction [35]. Although diversity seems to decrease following vaccine introduction, it is unknown whether the observed trends will be sustained in the long-term, especially in African settings where pre-vaccine genotype diversity is high.

Phylogenetic analysis of genotype G1 revealed circulation of strains of lineages G1-I and G1-II while the P [8] strains formed three distinct clusters revealing circulation of strains of lineages P [8]–I, P [8]-III and P [8]-IV. The Kenyan strains were closely related to other global strains as they segregated together. G1-I and P [8]-III were the predominant lineages for both pre and post-vaccine strains. These lineages were distinct from the Rotarix vaccine lineages showing that the strains were distantly related to the vaccine strain. Similar findings were observed in data collected before and after vaccine introduction in Eastern and Southern Africa countries [7]. Although there were no lineage replacements after vaccine introduction, the genetic diversity between pre and post-vaccine strains, may suggest normal genetic fluctuations or an effect of the vaccine. Interestingly, a single case of vaccine-derived strain was observed in a sample isolated from a child in Kibera, who had received the first dose of the Rotarix vaccine. Phylogenetically, the post-vaccine strain clustered with the Rotarix vaccine, in G1-I and P [8]-I lineages, and showed sequence identities of 100% to the vaccine strain. These clusters also contained previously vaccine-derived strains isolated in Brazil, Korea, and Japan. Acute gastroenteritis, caused by vaccine shedding or horizontal transmissions of the vaccine strain have been reported at a higher rate in other countries [48–50]. Further analysis will be necessary to determine whether the observed vaccine strain was as a result of vaccine shedding or horizontal transmission event.

This study provides substantial epidemiological information on changes in distribution and genetic diversity of RVA genotypes in Kenya. Some limitations of this study included fewer surveillance sites and unequal distribution of the number of samples among the sites, potentially underestimating the type of genotypes circulating in the post-vaccine period. However, it is important to consider that there was an overall decline in rotavirus circulation in the post-vaccine era, hence the fewer specimens and less diversity could be a reflection of less rotavirus circulation [14, 15]. Additionally, since this is an ecological study, the changes in distribution and diversity of genotypes in the post-vaccine era cannot directly be attributed to vaccine introduction. Furthermore, due to unsuccessful sequencing and/or contig assembly only two thirds of the total RVA positive samples were fully genotyped. Lastly these findings may not be generalized to the whole country because analysis was based on rotavirus cases observed in health facilities in three counties only.

## Conclusion

In conclusion, we highlight the importance of monitoring the transition in the prevalence of genotypes for a better understanding of the performance of the currently available vaccines. The emergence of the fully heterotypic G2P [4] and partially heterotypic G3P [8] genotypes after vaccine introduction raises questions about the epidemiological dynamics following vaccine introduction. Previous analysis in Kenya showed that the vaccine had a significant impact on G1P [8] and non-significant G2P [4] (although with limited statistical power) [14], hence, continuous monitoring of the circulating genotypes in the post-vaccine era is necessary. Our findings also highlight existence of considerable variation and genetic diversity within and between Kenyan pre and post-vaccine strains. One strain identified in Kenya, was closely related to the Rotarix® vaccine strain, likely representing shedding or horizontal transmission of the vaccine strain. Additionally, continued surveillance of the genetic characteristics of circulating RVA strains is recommended to obtain a full view of the long-term effects of vaccine introduction. Since immunity to RVA involves immune responses conferred by genes other than the commonly studied P and G genes, vaccine effectiveness might be challenged by changes occurring on non-capsid genes. It is therefore recommended that full genome analysis of genotypes collected in different time or geographic regions be conducted to improve understanding of their evolutionary profile during the post-vaccine introduction period.

## Supplementary information

**Additional file 1: Supplementary Figure 1**; Geographical boundaries and location of the health facilities participating in the rotavirus genotype surveillance program represented by the diamond shapes. Green – Siaya County Referral Hospital, blue – Lwak Mission Hospital, red – Tabitha Clinic and orange – Kilifi County Hospital.

**Additional file 2: Supplementary Table 1;** Frequency of partially typed G and P genotypes. Gx and P [x] were unsuccessfully typed due to failure in sequencing and/or contig assembly.

**Additional file 3: Supplementary Table 2;** GenBank accession numbers of all VP7 G gene sequences.

**Additional file 4: Supplementary Table 3;** GenBank accession numbers of all VP4 P gene sequences.

## Data Availability

Partial sequences for the VP7 and VP4 genes reported in this work have been deposited to GenBank database: https://www.ncbi.nlm.nih.gov/genbank/. Sequence accession numbers for the VP7 and VP4 genes are indicated in supplementary Table 2 and supplementary Table 3, respectively. The datasets used and analyzed during this study are available from the Harvard Dataverse: 10.7910/DVN/0VQ2OK. Public access to the data is restricted. Users who wish to access and use the data should send a request to the KEMRI Wellcome Trust Research Programme data governance committee, which can be contacted by emailing: dgc@kemri-wellcome.org.

## Abbreviations

RVA: Rotavirus group A
WHO: World Health Organization
AGE: Acute Gastroenteritis
VP: Viral protein
RIPEK: Rotavirus Immunization Programme Evaluation in Kenya
