# Supplementary figures and images for: Rotavirus group A genotype circulation patterns across Kenya before and after nationwide vaccine introduction, 2010–2018
Source: BMC Infect Dis. 2020 Jul 13;20:504. doi: 10.1186/s12879-020-05230-0 (PMC7359451; doi:10.1186/s12879-020-05230-0)

Supplementary Figure 1

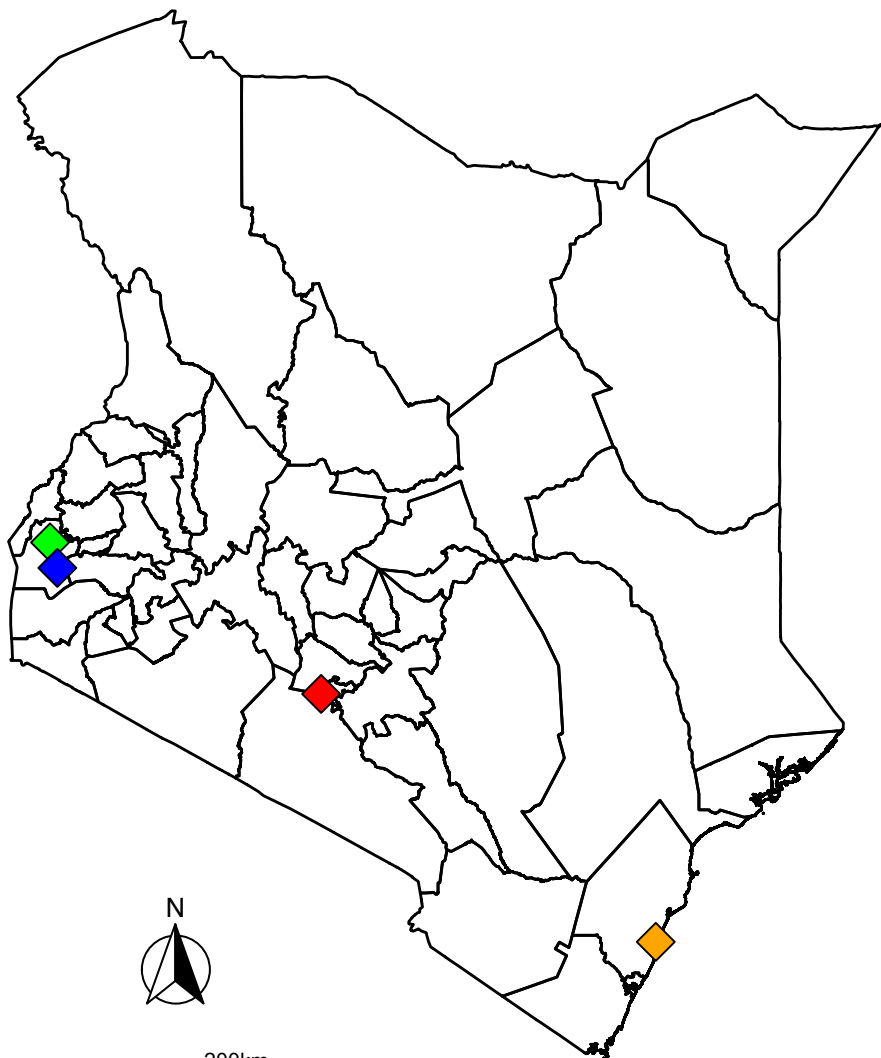

Supplement: Supplementary file 1 — Additional file 1: Supplementary Figure 1; Geographical boundaries and location of the health facilities participating in the rotavirus genotype surveillance program represented by the diamond shapes. Green – Siaya County Referral Hospital, blue – Lwak Mission Hospital, red – Tabitha Clinic and orange – Kilifi County Hospital. [file 12879_2020_5230_MOESM1_ESM.pdf]
