# Supplementary material for: Rotavirus group A genotype circulation patterns across Kenya before and after nationwide vaccine introduction, 2010–2018
Source: BMC Infect Dis. 2020 Jul 13;20:504. doi: 10.1186/s12879-020-05230-0 (PMC7359451; doi:10.1186/s12879-020-05230-0)
Supplement: Supplementary file 2 — Additional file 2: Supplementary Table 1; Frequency of partially typed G and P genotypes. Gx and P [x] were unsuccessfully typed due to failure in sequencing and/or contig assembly. [file 12879_2020_5230_MOESM2_ESM.pdf]

Supplementary Table 1

Genotype	2010		2011		2012		2013		2014		2015		2016		2017		2018		Total	
	n	%	n	%	n	%	n	%	n	%	n	%	n	%	n	%	n	%	n	%
G1P[x]	7	28.0	3	37.5	1	6.7	5	31.3	2	20.0	3	21.4	1	25.0	0	0.0	3	18.8	25	22.1
G2P[x]	0	0.0	0	0.0	0	0.0	0	0.0	1	10.0	0	0.0	0	0.0	1	20.0	0	0.0	2	1.8
G3P[x]	0	0.0	0	0.0	0	0.0	3	18.8	4	40.0	1	7.1	2	50.0	1	20.0	3	18.8	14	12.4
G8P[x]	4	16.0	0	0.0	2	13.3	0	0.0	0	0.0	0	0.0	0	0.0	0	0.0	0	0.0	6	5.3
G9P[x]	1	4.0	1	12.5	1	6.7	0	0.0	0	0.0	1	7.1	0	0.0	0	0.0	0	0.0	4	3.5
G12P[x]	4	16.0	1	12.5	2	13.3	0	0.0	0	0.0	0	0.0	0	0.0	1	20.0	2	12.5	10	8.8
G29P[x]	1	4.0	0	0.0	0	0.0	0	0.0	0	0.0	0	0.0	0	0.0	0	0.0	0	0.0	1	0.9
GxP[8]	5	20.0	3	37.5	5	33.3	6	37.5	3	30.0	9	64.3	0	0.0	1	20.0	4	25.0	36	31.9
GxP[4]	3	12.0	0	0.0	4	26.7	2	12.5	0	0.0	0	0.0	1	25.0	1	20.0	4	25.0	15	13.3
Total	25		8		15		16		10		14		4		5		16		113	
