# Supplementary material for: Rotavirus group A genotype circulation patterns across Kenya before and after nationwide vaccine introduction, 2010–2018
Source: BMC Infect Dis. 2020 Jul 13;20:504. doi: 10.1186/s12879-020-05230-0 (PMC7359451; doi:10.1186/s12879-020-05230-0)
Supplement: Supplementary file 3 — Additional file 3: Supplementary Table 2; GenBank accession numbers of all VP7 G gene sequences. [file 12879_2020_5230_MOESM3_ESM.pdf]

Supplementary table 2

GenBank accession numbers of all VP7 G gene sequences

MH402630	MH402616	MH402431	MH402303	MN194502	MH402228	MH402204	MH402731	MH402190
MH402179	MH402639	MH402105	MH402336	MH402692	MH402515	MH402124	MH402728	MH402382
MH402670	MH402436	MH402195	MH402347	MH402693	MH402283	MH402238	MH402333	MH402122
MH402528	MH402624	MH402219	MH402337	MH402713	MH402385	MH402696	MH402746	MH402193
MH402181	MH402617	MH402202	MH402350	MH402632	MH402525	MH402276	MH402335	MH402212
MH402628	MH402695	MH402218	MH402317	MH402619	MH402142	MH402271	MH402376	MH402194
MH402689	MH402452	MH402203	MH402292	MH402408	MH402180	MH402423	MH402729	MH402104
MH402389	MH402774	MH402215	MH402322	MH402656	MN194417	MN194416	MH402334	MH402191
MH402653	MH402722	MH402196	MH402312	MH402416	MH402095	MH402701	MH402753	MH402210
MH402675	MH402775	MH402300	MH402357	MH402627	MN194423	MH402279	MH402747	MH402189
MH402644	MH402116	MH402192	MH402368	MN194493	MN194500	MH402216	MH402773	MH402456
MH402676	MH402776	MH402213	MH402346	MH402229	MN194421	MN194412	MH402748	MH402768
MH402654	MH402626	MH402422	MH402342	MH402715	MN194428	MH402112	MH402777	MH402440
MH402420	MH402663	MH402266	MH402373	MH402633	MH402284	MH402217	MH402752	MH402438
MH402679	MH402618	MH402384	MH402313	MH402411	MN194422	MH402243	MH402749	MH402439
MH402677	MH402723	MH402262	MH402359	MH402287	MH402516	MH402201	MH402750	MH402441
MH402629	MH402661	MN194487	MH402360	MH402079	MH402523	MH402254	MH402751	MH402310
MH402678	MH402642	MH402268	MH402321	MH402414	MH402517	MH402275	MH402732	MH402457
MH402641	MH402643	MH402277	MH402370	MH402395	MH402129	MH402272	MH402739	MH402704
MH402706	MH402394	MH402265	MH402351	MN194494	MH402080	MH402088	MH402740	MH402315
MH402648	MH402637	MH402383	MH402325	MH402291	MH402130	MH402205	MH402741	MH402718
MH402657	MH402100	MH402299	MH402374	MH402714	MH402131	MH402121	MH402742	MH402719
MH402680	MH402662	MH402106	MH402327	MH402409	MH402132	MH402697	MH402733	MH402606
MH402649	MH402087	MH402417	MH402330	MH402103	MH402521	MN194504	MN194381	MH402182
MH402658	MH402655	MH402273	MH402339	MH402306	MH402437	MH402248	MH402734	MH402772
MH402659	MH402510	MH402118	MH402361	MH402620	MH402518	MH402258	MH402735	MH402614
MH402631	MH402399	MH402207	MH402328	MH402589	MH402133	MH402220	MH402736	MH402726
MH402671	MH402724	MH402700	MH402340	MH402290	MH402134	MH402699	MH402744	MH402602
MH402600	MH402527	MH402197	MH402358	MN194429	MH402135	MH402698	MH402754	MH402720
MH402102	MH402396	MH402286	MH402354	MH402412	MH402136	MH402267	MH402743	MH402672
MH402650	MH402148	MH402107	MH402331	MH402392	MH402522	MH402113	MH402755	MH402635
MH402449	MH402302	MH402214	MH402349	MN194410	MH402096	MH402278	MH402737	MH402413
MH402673	MH402169	MH402250	MN194406	MH402450	MH402781	MH402255	MH402745	MH402660
MH402587	MH402304	MH402208	MH402356	MH402610	MH402519	MH402260	MH402778	MH402621
MH402117	MH402400	MH402199	MH402353	MH402167	MH402756	MH402281	MH402738	MH402101
MH402603	MH402149	MH402246	MH402329	MH402230	MH402757	MH402282	MK434749	MH402512
MH402607	MH402126	MH402245	MH402345	MH402770	MH402186	MH402256	MK434565	MH402687
MH402588	MH402150	MH402235	MH402343	MH402231	MH402520	MH402779	MK434616	MH402415
MN194491	MH402184	MH402251	MH402311	MH402611	MH402758	MH402200	MK434580	MH402424
MH402608	MN194420	MH402252	MH402318	MH402645	MH402425	MH402612	MK434566	MH402721
MN194501	MH402174	MN194414	MH402369	MH402646	MH402759	MH402239	MK434768	MH402615
MH402308	MN194425	MH402125	MH402355	MH402085	MH402760	MH402114	MN194408	MH402622
MH402593	MH402171	MH402432	MH402341	MH402232	MH402139	MH402115	MN194448	MH402240
MH402609	MH402185	MH402257	MH402323	MH402640	MH402761	MH402144	MN194456	MH402623
MN194503	MH402224	MH402263	MH402362	MH402428	MH402097	MN194413	MN194449	MN194452
MH402664	MH402198	MH402253	MH402352	MH402444	MH402137	MH402707	MN194463	MN194470
MH402651	MN194418	MH402108	MH402348	MH402446	MH402524	MH402709	MN194405	MN194469

MH402604	MH402301	MH402225	MH402379	MH402140	MH402098	MH402674	MN194457	MN194461
MH402716	MH402288	MH402236	MH402371	MH402445	MH402285	MH402710	MN194495	MN194445
MN194411	MH402233	MH402123	MH402314	MH402447	MH402762	MH402401	MN194453	MN194446
MN194492	MH402127	MH402244	MH402316	MH402702	MH402089	MH402305	MN194505	MN194385
MH402690	MH402727	MH402259	MH402326	MH402429	MH402090	MH402708	MN194465	MN194447
MH402652	MH402514	MH402280	MH402380	MN194488	MH402763	MH402264	MN194458	MN194394
MH402711	MH402128	MH402269	MH402338	MN194497	MH402764	MH402433	MN194440	MN194437
MH402453	MH402172	MH402242	MH402319	MH402086	MH402769	MH402206	MN194379	MN194393
MH402605	MH402146	MH402227	MH402344	MH402430	MH402451	MH402638	MN194459	MN194434
MH402309	MN194419	MH402261	MH402363	MH402454	MH402526	MH402421	MN194392	MN194464
MN194489	MH402145	MH402109	MH402372	MH402458	MH402091	MH402434	MN194460	MN194468
MH402725	MH402170	MH402119	MH402332	MN194499	MH402092	MH402594	MN194454	MN194467
MH402634	MN194490	MH402110	MH402365	MH402187	MH402138	MH402665	MN194450	MN194378
MH402691	MH402397	MH402241	MH402320	MN194498	MH402093	MH402601	MN194390	MN194435
MH402712	MN194424	MH402120	MH402378	MN194496	MH402426	MH402668	MN194444	MN194432
MH402717	MN194426	MH402249	MH402366	MH402455	MH402141	MH402613	MN194443	MN194484
MH402406	MN194427	MH402111	MH402367	MH402234	MH402765	MH402435	MN194442	MN194485
MH402694	MN194441	MH402247	MH402381	MN194415	MH402448	MN194384	MN194466	MN194462
MH402410	MN194383	MH402274	MH402375	MH402188	MH402427	MH402771	MN194471	MH402393
MH402289	MH402173	MH402209	MH402377	MN194486	MH402094	MH402703	MN194455	MH402767
MH402407	MH402147	MH402270	MH402324	MH402211	MH402766	MH402625	MN194451	MK434596
MH402780	MH402151	MH402237	MH402364	MH402183	MK434705	MK434804	MN194374	MK434790
MK434786	MN194472	MK434744	MK434821	MK434757	MK434644	MK434801	MK434823	MN194375
MK434635	MK434701	MK434745	MK434727	MK434845	MK434775	MK434654	MK434631	MN194404
MK434685	MK434623	MK434742	MK434657	MK434824	MK434787	MK434679	MK434788	MN194388
MK434649	MK434735	MK434743	MK434722	MK434572	MK434776	MK434816	MK434717	MN194506
MK434728	MK434687	MK434681	MK434643	MK434830	MK434671	MK434803	MK434698	MN194377
MK434636	MK434689	MK434759	MK434627	MK434602	MK434777	MK434642	MK434832	MK434766
MK434650	MK434700	MK434677	MK434752	MK434573	MK434793	MK434624	MK434670	MK434668
MK434646	MK434736	MK434606	MK434822	MK434828	MK434754	MK434802	MK434651	MK434672
MK434704	MK434690	MK434568	MK434665	MK434846	MK434833	MK434798	MK434827	MK434713
MK434641	MK434613	MK434594	MK434628	MK434796	MK434706	MK434780	MK434729	MK434593
MK434640	MK434694	MK434608	MK434753	MK434834	MK434707	MK434781	MK434737	MK434664
MK434633	MK434588	MK434686	MK434666	MK434829	MK434708	MK434653	MK434712	MK434620
MK434732	MK434688	MK434582	MK434629	MK434587	MK434709	MK434785	MK434711	MK434621
MK434637	MK434792	MK434583	MK434630	MK434612	MK434710	MK434784	MK434844	MK434680
MK434778	MK434771	MK434609	MK434741	MK434726	MK434617	MK434818	MK434797	MK434718
MK434647	MK434699	MK434599	MK434740	MK434751	MK434600	MK434618	MK434634	MK434714
MK434638	MK434695	MK434603	MK434738	MK434576	MK434755	MK434703	MK434730	MK434716
MK434795	MK434696	MK434584	MK434673	MK434767	MK434570	MK434715	MK434747	MK434772
MK434622	MK434697	MK434569	MK434758	MK434577	MK434590	MK434683	MK434805	MK434765
MK434648	MK434782	MK434611	MK434674	MK434604	MK434574	MK434682	MK434601	MK434836
MK434663	MK434693	MK434585	MK434791	MK434598	MK434579	MK434847	MK434691	MK434814
MK434799	MK434667	MK434760	MK434748	MK434571	MK434575	MK434819	MK434806	MN194387
MK434800	MK434723	MK434763	MK434678	MK434578	MK434769	MK434820	MK434615	MK434597
MK434656	MK434655	MK434763	MK434719	MK434659	MK434586	MK434817	MK434808	MK434605
MK434676	MK434652	MK434770	MK434567	MK434756	MK434591	MK434625	MK434734	MK434813

MK434794	MK434750	MK434761	MK434645	MK434826	MK434592	MK434626	MK434783	MK434811
MK434731	MK434774	MK434839	MK434660	MK434831	MK434675	MN194402	MN194389	MK434842
MK434632	MK434614	MK434815	MK434733	MK434825	MK434607	MN194396	MN194436	MK434838
MK434779	MK434724	MK434789	MK434746	MK434589	MK434595	MN194398	MN194507	MK434843
MK434639	MK434692	MK434809	MK434661	MK434837	MK434721	MN194479	MN194391	MK434807
MK434658	MK434720	MK434764	MK434662	MK434812	MK434610	MN194474	MN194380	MK434841
MK434725	MK434702	MK434810	MK434669	MN194373	MK434581	MN194403	MN194438	MK434684
MK434835	MK434773	MK434762	MK434619	MK434840	MK434739	MN194481	MN194386	MN194382
MN194476	MN194483	MN194430	MN194477	MN194399	MN194480	MN194433	MN194431	MN194397
MN194400	MN194475	MN194401	MN194509	MN194478	MN194473	MN194482	MN194409	MN194439
MN194376	MN194395	MN194407	MN194508					
