# Supplementary material for: Rotavirus group A genotype circulation patterns across Kenya before and after nationwide vaccine introduction, 2010–2018
Source: BMC Infect Dis. 2020 Jul 13;20:504. doi: 10.1186/s12879-020-05230-0 (PMC7359451; doi:10.1186/s12879-020-05230-0)
Supplement: Supplementary file 4 — Additional file 4: Supplementary Table 3; GenBank accession numbers of all VP4 P gene sequences. [file 12879_2020_5230_MOESM4_ESM.pdf]

Supplementary table 3

GenBank accession numbers of all VP4 G gene sequences

MH403257	MH403192	MH403266	MH403487	MH403441	MK434361	MH402811	MH403501	MH403237
MK434500	MH403405	MH402930	MH403485	MH403338	MK434454	MH402818	MH403534	MH403300
MK434400	MH403178	MH402844	MH402968	MH403339	MK434479	MH402812	MH403474	MH403435
MK434397	MH403171	MH403411	MH403480	MH403232	MK434485	MH402838	MH403517	MH403301
MK434402	MH403172	MH403542	MH402973	MN194350	MK434435	MH402866	MH403533	MH403302
MK434383	MH403216	MH403273	MH402994	MH403200	MK434381	MH402862	MH403514	MH403303
MK434399	MH403004	MH403187	MH402986	MH403317	MK434447	MN194275	MH403502	MH403438
MK434403	MN194345	MH403446	MH402983	MH403156	MK434416	MH402863	MH403493	MH403304
MK434407	MH403176	MH403412	MH402982	MN194360	MK434392	MH403164	MH403524	MH403423
MK434553	MH403179	MH403365	MH402984	MH403000	MK434424	MH402841	MH403475	MH403440
MK434391	MH403180	MH402928	MH402970	MH403424	MK434425	MH403361	MH403471	MH403305
MK434404	MH403207	MH403413	MH402971	MN194347	MK434436	MH402819	MH403531	MH403217
MK434396	MH403357	MH402837	MH402972	MH403428	MK434455	MH402820	MH403510	MH403227
MK434458	MN194357	MH402788	MH402974	MH403236	MK434382	MH403277	MH403520	MH403401
MK434384	MH403181	MH402876	MH402987	MH403228	MK434350	MH402821	MH403521	MH403377
MK434398	MH403241	MH402873	MH402991	MH403225	MK434426	MN194274	MH403530	MH403231
MK434395	MH403185	MH403366	MN194266	MH403155	MK434417	MH402881	MH403511	MH403201
MK434387	MH403222	MH402921	MH402989	MH403318	MK434356	MH403362	MH403503	MH403255
MK434388	MH403246	MH403414	MH402980	MH402953	MK434444	MH402791	MH403496	MN194346
MK434516	MH403206	MH403450	MH402993	MH403214	MK434393	MH402950	MH403518	MH403306
MK434518	MH403385	MH403380	MH402975	MN194348	MK434511	MH402867	MH403538	MH403101
MK434524	MH403182	MH403382	MH402992	MH402854	MK434427	MH402813	MH403529	MH403437
MK434389	MH403559	MH402833	MH402976	MH403319	MK434428	MH402830	MH403497	MH403307
MK434394	MH403342	MH403161	MH402981	MH403215	MK434484	MH402822	MH403506	MH403221
MK434525	MH403386	MH403267	MH402990	MH403320	MK434378	MH402847	MH403505	MH403218
MK434385	MH403343	MH402952	MH402988	MH403006	MK434429	MH402852	MH403494	MH403229
MK434478	MH403387	MH403268	MH402985	MH403340	MK434406	MH402856	MH403476	MH403220
MK434526	MH403203	MH402834	MH402977	MH403544	MK434430	MH402955	MH403495	MH403238
MK434482	MH403388	MH403398	MH402995	MH403419	MK434550	MH402793	MH403526	MH403335
MK434405	MH403212	MH402882	MH402979	MH403321	MK434357	MH402883	MH403492	MH403173
MK434453	MH403324	MN194352	MH402978	MH403005	MK434431	MH402875	MH403484	MH403346
MK434411	MH403389	MH403269	MK434376	MH402931	MK434432	MH402845	MH403477	MH403166
MK434374	MH403430	MH403270	MK434349	MH403451	MK434551	MH402817	MN194292	MH403308
MK434373	MH403396	MH402855	MK434336	MH402926	MK434552	MH402933	MH403478	MH403239
MK434365	MH403390	MH403399	MK434360	MN194355	MK434549	MH403381	MH403481	MH403336
MK434366	MH403391	MH403276	MK434355	MH403193	MK434498	MH402839	MH403482	MH403253
MK434367	MH403425	MH403367	MN194325	MH402932	MK434351	MH403174	MH403528	MH403347
MK434368	MH402935	MH403368	MN194339	MH403330	MK434337	MH403363	MH403479	MH403439
MK434563	MH403350	MH403442	MN194311	MH403378	MK434362	MH402831	MH403527	MH403309
MK434369	MH403433	MN194353	MN194335	MH403233	MK434352	MN194273	MH403525	MH403310
MK434370	MH402937	MH403369	MN194312	MH402865	MK434338	MH403250	MH403489	MH403337
MK434372	MH402936	MH403271	MN194291	MH403458	MK434433	MH402927	MH403483	MH403431

MK434408	MH403397	MH403370	MN194313	MH403202	MK434386	MH402956	MH403490	MH403213
MK434519	MH402938	MH403332	MN194343	MH402794	MK434437	MH402814	MH403491	MH403311
MK434520	MH403550	MH403421	MN194336	MH402877	MK434438	MH402815	MH403508	MN194349
MK434371	MH402947	MH403400	MN194314	MH402843	MK434434	MH403364	MH403504	MH403249
MK434515	MH403358	MH403420	MN194315	MN194358	MK434439	MH403409	MH403535	MH403312
MK434534	MH402939	MH403371	MN194333	MH402859	MK434364	MH402919	MH403499	MH403313
MK434521	MH402948	MN194351	MN194316	MH403175	MK434410	MH402961	MH403540	MH403353
MK434377	MH403392	MH403372	MN194264	MH402868	MK434343	MH402842	MH403498	MH403314
MK434527	MH402940	MH403403	MN194277	MH402959	MK434339	MN194371	MH403539	MH403560
MK434528	MH403351	MH403278	MN194278	MH402878	MK434344	MH402929	MH403523	MH403254
MK434530	MH403393	MH403404	MN194326	MH403447	MK434363	MH402846	MH403541	MH403462
MK434531	MH403183	MH403406	MN194327	MH402951	MK434340	MH402832	MH403537	MH403167
MK434529	MH403551	MH403373	MN194337	MH402962	MK434375	MH403415	MH403512	MH403426
MK434517	MH403552	MH403279	MN194279	MH402840	MK434341	MH402874	MH402996	MH403224
MN194262	MH402941	MH403280	MN194328	MH403322	MK434358	MH403186	MH402967	MH403315
MK434522	MH403432	MH403333	MN194317	MH402954	MK434353	MH402848	MH403486	MH403427
MK434532	MH403394	MH403374	MN194287	MH402857	MK434345	MH403410	MH402969	MH403316
MK434533	MH403553	MH403375	MN194318	MH402957	MK434468	MK434466	MH403162	MN194366
MK434523	MH402949	MH403251	MN194319	MH402864	MK434471	MK434457	MH402799	MN194301
MK434538	MH402942	MH403281	MN194320	MH402963	MK434461	MK434465	MH403272	MN194304
MK434536	MH403359	MH403282	MN194329	MH402960	MK434514	MK434476	MH402823	MN194309
MK434540	MH403416	MH403468	MN194321	MH402885	MK434561	MK434502	MH403408	MN194272
MK434539	MH403325	MH403283	MN194338	MH403188	MK434440	MK434462	MH402872	MN194300
MK434564	MH403395	MH403284	MN194340	MH402795	MK434440	MK434554	MH403402	MN194368
MK434543	MH403326	MH403285	MN194322	MH402803	MK434469	MK434449	MH402998	MN194299
MK434537	MH403360	MH403286	MN194323	MH403189	MK434470	MK434442	MH403001	MN194302
MK434541	MH403327	MH403287	MN194330	MH403460	MK434544	MK434450	MH403547	MN194297
MK434359	MH402944	MH403288	MN194331	MH402858	MK434508	MK434452	MH403461	MN194303
MK434342	MH403429	MH403289	MN194280	MH403197	MK434504	MK434501	MH403208	MN194365
MK434348	MH403384	MH403290	MN194332	MH403348	MK434535	MK434463	MH403191	MN194344
MK434483	MH403417	MH403376	MN194281	MH402997	MK434513	MK434467	MH403240	MK434495
MK434496	MH402945	MH403291	MN194282	MH402934	MK434545	MK434460	MH403234	MK434490
MK434542	MH402943	MH403329	MN194283	MH402835	MK434562	MK434481	MN194362	MK434491
MN194276	MH403464	MH403292	MN194284	MH402869	MK434472	MK434487	MH403209	MK434548
MN194261	MH402946	MH403293	MN194334	MH403323	MK434499	MK434346	MN194359	MK434415
MN194342	MH403349	MH403383	MN194341	MH402886	MK434448	MK434421	MH403002	MK434506
MN194288	MH403344	MH403294	MN194324	MH402918	MN194305	MK434556	MH403459	MK434507
MN194370	MH403345	MH403334	MN194263	MH402789	MN194270	MK434445	MH403355	MK434512
MH402923	MH403328	MH403295	MN194285	MH402798	MN194306	MK434497	MH403455	MK434510
MH403230	MH403470	MH403196	MN194286	MH403379	MN194310	MK434503	MH403168	MK434451
MH402807	MH403466	MH403296	MN194363	MH402870	MN194372	MK434414	MH403177	MK434446
MH403163	MH403543	MH403297	MN194364	MH402790	MN194268	MK434347	MH403223	MK434509

MH403331	MH403536	MH403190	MK434379	MH403354	MN194267	MK434559	MH403219	MK434380
MH402782	MH403465	MH403242	MK434473	MH402965	MN194293	MK434474	MH403243	MK434423
MH403445	MH403472	MH403463	MK434413	MH402958	MN194296	MK434475	MH403247	MK434412
MH402828	MH403519	MH403235	MK434505	MH403546	MN194289	MK434488	MN194354	MK434560
MH402783	MH403516	MH403256	MK434477	MH402964	MN194269	MK434557	MH403210	MK434401
MH402784	MH403473	MH403252	MK434418	MH403341	MN194265	MK434489	MH403244	MK434443
MH402829	MH403500	MH403434	MK434456	MH402966	MN194298	MK434422	MH403356	MK434409
MH403352	MH403157	MH403436	MK434419	MH402797	MN194294	MK434555	MH403169	MH403457
MH402785	MH403507	MN194361	MK434441	MH402879	MN194307	MK434480	MH403158	MH403248
MH402808	MH403513	MH403165	MK434459	MH402836	MN194271	MK434547	MN194356	MH403245
MH402802	MH403515	MH403298	MK434354	MH402884	MN194290	MK434558	MH403003	
MH402880	MH403509	MH403226	MK434420	MH402871	MN194369	MK434492	MH403211	
MH402809	MH403522	MH403422	MK434546	MH402786	MN194367	MK434493	MH403184	
MH402925	MH403488	MH403204	MK434486	MH402801	MN194295	MK434390	MH403170	
MH402810	MH403532	MH403299	MK434464	MH403448	MN194308	MK434494	MH403205	
